# Outcome of lung transplant recipients infected with SARS-CoV-2/Omicron/B.1.1.529: a Nationwide German study

**DOI:** 10.1007/s15010-022-01914-8

**Published:** 2022-09-09

**Authors:** Nikolaus Kneidinger, Matthias Hecker, Vasiliki Bessa, Ina Hettich, Alexandra Wald, Sabine Wege, Anna-Barbara Nolde, Maike Oldigs, Zulfiya Syunyaeva, Heinrike Wilkens, Jens Gottlieb

**Affiliations:** 1 Department of Medicine V, Comprehensive Pneumology Center (CPC-M), Member of the German Center for Lung Research (DZL), University Hospital, LMU Munich, Marchioninistrasse 15, 81377 Munich, Germany; 2 Department of Internal Medicine, Universities of Giessen and Marburg Lung Center (UGMLC), Member of the German Center for Lung Research (DZL), Justus-Liebig University, Giessen, Germany; 3 Department of Pulmonary Medicine, West German Center for Lung Transplantation, University of Duisburg-Essen, Essen, Germany; 4 Department of Pneumology, Medical Center—University of Freiburg, Faculty of Medicine, University of Freiburg, Freiburg, Germany; 5 Department of Respiratory Medicine, University of Leipzig, Leipzig, Germany; 6 Department of Pneumology and Critical Care Medicine, Translational Lung Research Center (TLRC), German Center for Lung Research (DZL), Thoraxklinik at the University of Heidelberg, Heidelberg, Germany; 7 Department of Respiratory Medicine, Eppendorf University Hospital, Hamburg, Germany; 8 Department of Pulmonology, Airway Research Center North, Member of the German Center for Lung Research (DZL), LungenClinic Grosshansdorf, Großhansdorf, Germany; 9 Department of Pediatric Respiratory Medicine, Immunology and Critical Care Medicine and Cystic Fibrosis Center, Charité Universitätsmedizin Berlin, Berlin, Germany; 10 Department of Internal Medicine V, Pulmonology, Allergology, Respiratory Intensive Care Medicine, Saarland University, Homburg Saar, Germany; 11 Department of Respiratory Medicine, Hannover Medical School, German Center for Lung Research (DZL), Biomedical Research in Endstage and Obstructive Lung Disease Hannover (BREATH), Hannover, Germany

**Keywords:** Omicron, COVID-19, Transplantation, Immunosuppression, Lung

## Abstract

**Purpose:**

Coronavirus disease 2019 (COVID-19) caused by severe acute respiratory syndrome coronavirus type 2 (SARS-CoV-2) is currently the major threat for immunocompromised individuals. The course of COVID-19 in lung transplant recipients in the Omicron era remains unknown. The aim of the study was to assess outcome and associated factors in lung transplant recipients in a German-wide multicenter approach.

**Methods:**

All affected individuals from January 1st to March 20th, 2022 from 8 German centers during the Omicron wave were collected. Baseline characteristics and antiviral measures were associated with outcome.

**Results:**

Of 218 patients with PCR-proven SARS-CoV-2 infection 166 patients (76%) received any early (< 7 days) antiviral therapy median 2 (interquartile range 1–4) days after symptom onset. Most patients received sotrovimab (57%), followed by remdesivir (21%) and molnupiravir (21%). An early combination therapy was applied in 45 patients (21%). Thirty-four patients (16%) developed a severe or critical disease severity according to the WHO scale. In total, 14 patients (6.4%) died subsequently associated with COVID-19. Neither vaccination and antibody status, nor applied treatments were associated with outcome. Only age and glomerular filtration rate < 30 ml/min/1.73m^2^ were independent risk factors for a severe or critical COVID-19.

**Conclusion:**

COVID-19 due to Omicron remains an important threat for lung transplant recipients. In particular, elderly patients and patients with impaired kidney function are at risk for worse outcome. Prophylaxis and therapy in highly immunocompromised individuals need further improvement.

## Introduction

Coronavirus disease 2019 (COVID-19) caused by severe acute respiratory syndrome coronavirus type 2 (SARS-CoV-2) is currently the major threat for immunocompromised individuals. Lung transplant recipients are believed to be at particular risk for worse outcome due to the high dose of immunosuppressive drugs and the lung as the main organ affected by COVID-19 [1, 2].

Before the use of active immunization and other antiviral measures, 30-day mortality rates of lung transplant recipients with COVID-19 of 30–40% were reported [3, 4]. Later during the pandemic both hospitalization and mortality rates have declined, [5] which has been associated with vaccination rates [6] and early application of monoclonal antibodies (mAbs) [7]. However, mortality remains significant and long-term sequelae remain unknown.

At the end of 2021, the new variant of concern (VOC) Omicron (B.1.1.529) has become the dominant SARS-CoV-2 strain globally. High transmissibility due to the ability to evade SARS-CoV-2 immunity acquired by either vaccination or past infection [8, 9] and likely other intrinsic virological properties, like faster replication in the upper airways resulted in a dramatic increase of affected individuals and the largest wave since the beginning of the pandemic [10]. Yet, Omicron-infected individuals have significantly reduced odds of severe disease compared with individuals infected earlier with the VOC Delta (B.1.617.2) [11]. However, in patients requiring hospitalization disease severity seems to be comparable to Delta [12].

Furthermore, concomitantly to the appearance of Omicron a variety of antiviral treatments became available. The mAb sotrovimab with neutralizing activity against Omicron [13, 14] and the antiviral drug molnupiravir alone [15] or in combination with other antiviral measures have been widely accepted as standard of care particularly in unvaccinated and immunocompromised patients with COVID-19.

However, despite potentially milder disease and new antiviral measures the outcome of COVID-19 in lung transplant recipients remains unknown. In addition to the immune escape of Omicron, the antibody response after vaccination is frequently insufficient in immunocompromised individuals mitigating the beneficial effect of a complete primary series and booster doses of vaccines [16]. Furthermore, antiviral drugs and combinations thereof have not been studied in transplant recipients.

Therefore, the course of COVID-19 in lung transplant recipients in the Omicron era remains unknown. The aim of this study was to assess outcome and associated factors of patients after lung transplantation with COVID-19 in a German-wide initiative during the recent Omicron wave.

## Methods

All lung transplant recipients with polymerase chain reaction (PCR)-proven SARS-CoV-2 infection from January 1st to March 20th, 2022 from 8 German lung transplant centers (Berlin, Essen, Freiburg, Giessen, Hamburg, Hannover, Homburg, Leipzig and Munich) were included in the study. The study was approved by the central institutional ethics committee (Munich, Germany; project number 22-0078). This retrospective study was performed in accordance with the ethical guidelines of the 2000 Declaration of Helsinki and the standards of the 2008 Declaration of Istanbul.

In the beginning of January, Omicron (B.1.1.529) became the dominant VOC in Germany. To limit the analysis to patients with Omicron (B.1.1.529), lung transplant recipients with SARS-CoV-2 infection from January 1st to 31st were only included if SARS-CoV-2 VOC characterization was available and Omicron (B.1.1.529) detected. From 1^st^ of February on, when Omicron (B.1.1.529) was almost exclusively (> 98%) present in Germany all affected individuals were included, if no other VOC was detected. If Omicron sublineages BA.1 and BA.2 were not recorded, patients with SARS-CoV-2 infection before February 21^st^ were attributed to BA.1. Thereafter, patients were attributed to BA.2 according to the dominant occurrence of sublineages in Germany.

Baseline characteristics (age, sex, underlying disease, lung transplant procedure: bilateral, unilateral or combined organ transplantation and immunosuppressive regimen) and comorbidities (diabetes mellitus, obesity (defined as body mass index > 30 kg/m^2^), chronic lung allograft dysfunction (CLAD), chronic kidney disease (i.e., glomerular filtration rate (GFR) < 30 ml/min/1.73m^2^) and coronary artery disease were recorded. CLAD was defined according to the established criteria [17].

Furthermore, number and dates of previous vaccinations, type of vaccine, previous infections with SARS-CoV-2 and last available antibodies against Spike-protein (SARS-CoV-2-S) were recorded. A complete primary series of vaccination was defined as 3 doses of either mRNA-vaccines (BNT162b2/BioNTech/Pfizer, mRNA-1273/Moderna), 3 doses of ChAdOx1/AstraZeneca, combinations thereof or one dose Ad26.COV2.S/Johnson & Johnson a second dose of either mRNA COVID-19 vaccines. More vaccinations were defined as a complete primary series plus booster vaccination. SARS-CoV-2-S antibody status was classified in unknown, negative (binding antibody units (BAU) < 50/ml), low (BAU 50–250/ml) and positive (BAU > 250/ml).

Dates of symptom onset, SARS-CoV-2 infection proven by PCR and applied treatments, respectively, were recorded. Available antiviral treatments during the study periods were casirivimab/imdevimab, sotrovimab, remdesivir, molnupiravir and since the end of February nirmatrelvir/ritonavir. Antiviral treatments were regarded as early if started within less than 7 days after symptom onset. In the case of asymptomatic patients, the date of positive SARS-CoV-2 PCR was regarded as the date of disease onset. Other antiinflammatory treatments like dexamethasone and tocilizumab were recorded but not analyzed further due to their application only in advanced stages of disease.

COVID-19 severity was scored according to the WHO scale [18] with recording of the highest stage during follow-up after infection. In brief, mild disease was defined as constitutional symptoms without signs of pneumonia or respiratory failure. Moderate disease had signs of pneumonia without respiratory failure (blood oxygen saturation (SpO2) > 94%, no use of oxygen). Patients admitted for non-pulmonary manifestations were graded as moderate disease. Severe disease was defined as respiratory rate ≥ 30/min, SpO_2_ < 94%, use of oxygen or opacities > 50% on pulmonary imaging. Critical disease was defined as respiratory failure with need of mechanical ventilation support, presence of septic shock or multiple organ failure.

Follow-up after COVID-19 was recorded for at least 28 days or until death whichever occurred first.

### Statistics

Metric variables were expressed as medians and interquartile range (IQR). Univariate analyses were performed using the Mann–Whitney test for continuous variables and chi-square test for categorical variables. Binary logistic regression analyses were conducted with severe or critical course of disease as the dependent variable. The level of significance was set at < 0.10 for including variables identified by univariate analysis between groups.

## Results

In total, 218 patients were identified and included in the study. Patient characteristics are shown in Table 1. In brief, median age at time of COVID-19 was 56 (25, 75% percentile 45, 63) and 104 patients (48%) were female. Pre-existing CLAD was present in 53 individuals (24%) and comorbidities in descendent frequency were diabetes mellitus (*n* = 64, 29%), coronary artery disease (*n* = 35, 16%), chronic kidney disease (*n* = 32, 15%) and obesity (*n* = 12, 6%).

**Table 1:** Patient characteristics

Lung transplant recipients with SARS-CoV-2 infection *n* = 218	
Sex, *n* (%)	
Male	114 (52)
Female	104 (48)
Age at infection, median years (25, 75% percentile)	56 (45, 63)
Time after transplant, median years (25, 75% percentile)	4.0 (2.3, 8.0)
Lung transplant procedure, *n* (%)	
Bilateral	199 (91)
Unilateral	15 (7)
Combined	4 (2)
Underlying disease, *n* (%)	
Emphysema	58 (27)
Pulmonary vascular disease	10 (5)
Cystic fibrosis/bronchiectasis	40 (18)
Fibrosis/interstitial lung disease	80 (37)
Other	30 (14)
Pre-existing CLAD, *n* (%)	53 (24)
CLAD stage 1 and 2	38 (17)
CLAD stage 3 and 4	15 (7)
Phenotype BOS	34 (16)
Phenotype RAS/mixed	11 (5)
Phenotype other	8 (4)
Immunosuppression, *n* (%)	
Tacrolimus	189 (87)
Ciclosporine	28 (13)
Calcineurin-inhibitor free	1(0)
Purine antagonist	203 (93)
Proliferation signal inhibitor	35 (16)
Quadruple immunosuppression	22 (10)
Comorbidities, *n* (%)	
Chronic kidney disease (GFR < 30 ml/min/1.73m^2^)	32 (15)
Coronary artery disease	35 (16)
Diabetes with drug treatment	64 (29)
Obesity (body mass index > 30 kg/m^2^)	12 (6)
Days after last vaccination, median days (25, 75% percentile)	103 (77, 166)
Follow-up, median days (25, 75% percentile)	74 (59, 89)

Data are presented as number (*n*) and percentage (%) and median years (25, 75% percentile), respectively, as indicated. *BOS*, bronchiolitis obliterans syndrome; *CLAD*, chronic lung allograft dysfunction; *GFR*, glomerula filtration rate; *RAS*, restrictive allograft syndrome; *SARS-CoV-2*, severe acute respiratory syndrome coronavirus type 2

In 57 patients (26%), VOC was tested with confirmed Omicron BA.1; in 39 patients (68%) Omicron BA.2 in 18 patients (32%), respectively.

**Fig. 1:**
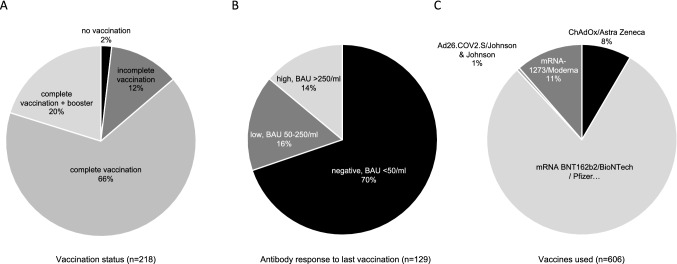
Vaccinations status (**A**), antibody response (**B**) and vaccines used (**C**) of the study cohort. List of abbreviations: BAU, binding antibody units

Vaccination status, antibody responses and vaccines used are shown in Fig. 1. A total of 188 patients (86%) had received a complete series of primary vaccines. Thereof, 44 patients (23%) received at least one booster vaccination. No vaccination was reported by 4 patients (2%). The most frequent vaccine was mRNA BNT162b2/BioNTech/Pfizer (80%). The majority of patients (70%) with known antibody status had a negative SARS-CoV-2-S antibody response. No patient with a negative antibody status had a low or positive antibody status in a previous assessment. No pre-exposure prophylaxis was used in patients.

**Fig. 2:**
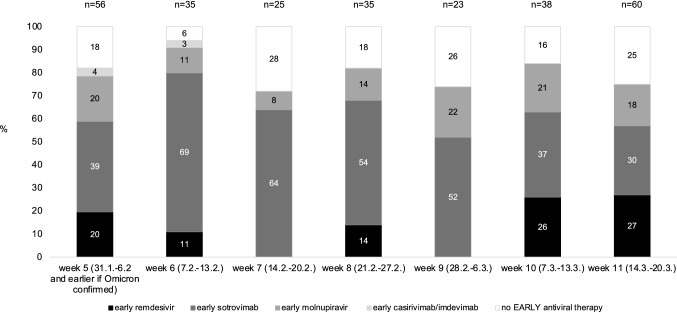
Early antiviral treatment during the study period. Percentages of applied antiviral treatments according to the week of the study are shown as stacked bars chart. The number of early treatments is provided above the bars

Any early antiviral therapy (i.e., < 7 days of symptom onset) was given in 166 patients (76%) median 2 (IQR 1, 4) days after symptom onset. Of the remaining 52 patients (24%) 28 patients (14%) received treatment ≥ 7 days (median 9 days, IQR 7, 12) and 24 patients (12%) received no antiviral treatment at all. A combination of early antiviral treatments was given in 45 patients (21%). As shown in Table 2 early sotrovimab was the most frequent treatment (*n* = 125, 57%) followed by remdesivir (*n* = 46, 21%) and molnupiravir (*n* = 46, 21%). The applied treatments over the study period are shown in Fig. 2. No change in preferred treatment modalities was noted during the study period.

**Table 2:** Treatment and outcome

Lung transplant recipients with SARS-CoV-2 infection *n* = 218	
Time to therapy, median days (25, 75% percentile)	2 (1, 4)
Hospital admission, *n* (%)	
Any admission during follow-up	92 (42)
Late admission	35 (16)
Previous SARS-CoV-2 infection, *n* (%)	5 (2)
Disease severity, *n* (%)	
Asymptomatic	18 (8)
Mild	137 (63)
Moderate	29 (13)
Severe	17 (8)
Critical	17 (8)
Death during follow-up, *n* (%)	13 (6)
COVID-19-related death during follow-up	12 (6)
Remdesivir, *n* (%)	57 (26)
Remdesivir < 7 days of symptom onset	46 (21)
Sotrovimab, *n* (%)	140 (64)
Sotrovimab < 7 days of symptom onset	125 (57)
Molnupiravir, *n* (%)	55 (25)
Molnupiravir < 7 days of symptom onset	46 (21)
Nirmatrelvir/Ritonavir, *n* (%)	1 (0)
Nirmatrelvir/Ritonavir < 7 days of symptom onset	0 (0)
Casirivimab/Imdevimab, *n* (%)	3 (1)
Casirivimab/Imdevimab < 7 days of symptom onset	3 (1)
Any antiviral therapy < 7 days of symptom onset, *n* (%)	166 (76)
Any combination therapy < 7 days of symptom onset, *n* (%)	45 (21)
Any triple antiviral therapy < 7 days of symptom onset, *n* (%)	13 (6)

Data are presented as number (*n*) and percentage (%) and median years (25, 75% percentile), respectively, as indicated. *COVID-19*, coronavirus disease 2019; *SARS-CoV-2*, severe acute respiratory syndrome coronavirus type 2

Disease severity is shown in Table 2. Most frequently, a mild course of disease (*n* = 137, 63%) was recorded. A total of 34 patients (16%) had a severe or critical course of disease. 15 patients (6.9%) died during follow-up and 14 deaths (6.4%) were attributed to COVID-19 median 27 (IQR 22, 33) days after disease onset.

In univariate regression analysis age at infection, time since lung transplantation and unilateral transplantation were associated with a severe or critical course of disease. Further associations were found for a GFR < 30 ml/min/1.73m^2^ and pre-existing CLAD. Immunosuppressive regime, vaccination and SARS-CoV-2-S antibody response and early antiviral therapies were not associated with a severe or critical course of disease (Table 3). In multivariate regression analysis, only age at infection (odds ratio (OR) 1.082, 95% CI 1.015–1.153; *p* = 0.015) and a GFR < 30 ml/min/1.73m^2^ (OR 3.175, 95% CI 1.278–7.884; *p* = 0.013) were associated with a severe or critical COVID-19 as shown in Table 3.

**Table 3:** Group comparison and regression analysis

Covariate	Group comparison		Univariate	Multivariate analysis	
	Lung transplant recipients with severe/critical COVID-19 *n* = 34	Lung transplant recipients with non-severe COVID-19 *n* = 184	*p* value	Odds ratio (95%-confidence interval)	*p* value
Female sex, *n* (%)	13 (38)	91 (49)	0.229		
Age at infection, median years (25, 75% percentile)	60 (53, 66)	55 (43, 63)	** < 0.001**	**1.082 (1.015 -1.153)**	**0.015**
Time after transplant, median years (25, 75% percentile)	6.9 (3.3, 11.4)	5.5 (2.1, 9.4)	0.026	1.028 (0.936–1.130)	0.559
Transplant procedure, *n* (%)					
Bilateral incl. combined	28 (82)	175 (95)	0.019	Reference	
Unilateral	6 (18)	9 (5)		1.770 (0.501–6.247)	0.375
Underlying disease, *n* (%)					
Emphysema and interstitial lung disease	28 (82)	110 (60)	0.066	Reference	
Cystic fibrosis /bronchiectasis / pulmonary vascular disease	2 (5)	48 (26)		0.744 (0.183–3.016)	0.679
Other	4 (12)	26 (14)		0.519 (0.078–3.459)	0.498
Comorbidities, *n* (%)					
Body mass index > 30 kg/m^2^	1 (3)	11 (6)	0.476		
Glomerular filtration rate < 30 ml/min/1.73m^2^	13 (38)	20 (11)	< 0.001	**3.175 (1.278–7.884)**	**0.013**
Diabetes	14 (41)	50 (27)	0.100		
Pre-existing chronic lung allograft dysfunction	13 (38)	40 (21)	0.039	1.266 (0.456–3.513)	0.651
Immunosuppression, n (%)					
Tacrolimus	28 (82)	161 (88)	0.608		
Ciclosporine	6 (18)	22 (12)			
Purine antagonist	34 (100)	169 (92)	0.226		
Proliferation signal inhibitor	5 (15)	30 (16)	0.816		
Variant era, *n* (%)					
BA.1 until 20–2-2022 or typing	22 (65)	114 (62)	0.761		
BA.2 from 21–2-2022 or typing	12 (35)	70 (38)			
vaccination status, n (%)					
none or incomplete vaccination	5 (15)	25 (13)	0.925		
complete vaccination ± booster	29 (85)	158 (87)			
Treatment, *n* (%)					
No early antiviral therapy	7 (21)	45 (25)	0.627		
Remdesivir < 7 days after symptom onset	6 (18)	40 (22)	0.706		
Sotrovimab < 7 days after symptom onset	21 (62)	104 (57)	0.790		
Molnupiravir < 7 days after symptom onset	8 (24)	38 (21)	0.706		
Casirivimab-Imdevimab < 7 days after symptom onset	0 (0)	3 (2)	0.453		
Any antiviral treatment < 7 days after symptom onset	27 (79)	139 (76)	0.627		
Combination antiviral treatment < 7 days after symptom onset	8 (24)	37 (20)	0.759		
Antibody response after last vaccination, *n* (%)					
Unknown	12 (35)	77 (42)	0.226		
Negative (BAU < 50/ml)	19 (56)	71 (39)			
Low (BAU 50–250/ml	2 (6)	19 (10)			
Positive (BAU > 250/ml	1 (6)	17 (9)			

In multivariate analysis bold indicates statistical significance (*p*< 0.05)

Data are presented as number (*n*) and percentage (%) and median years (25, 75% percentile), respectively, as indicated. *BAU*, binding antibody units; *COVID-19*, coronavirus disease 2019

## Discussion

In this multicenter national study during the Omicron (B.1.1.529) wave, one of six lung transplant recipients developed a severe or critical course of disease and COVID-19-associated mortality was 6.4%. Neither vaccination status and SARS-CoV-2-S antibody response, nor early antiviral treatments and combinations thereof seem to significantly impact the course of disease. Age and GFR < 30 ml/min/1.73m^2^ were independently associated with severe or critical COVID-19.

In contrast to previous reports of COVID-19 with other variants, the severity of disease in lung transplant recipients has decline; which is in line with previous reports on non-transplant individuals [11, 19, 20]. COVID-19-associated mortality has been reported of up to 30–40% in early single center studies and was 6.4% in our current analysis [3, 4]. Causes of more favorable course of COVID-19 with Omicron remain poorly understood. Omicron seems to have a tropism for the upper respiratory tract rather than the lower respiratory tract possibly leading to less COVID-19-associated pneumonias and therefore less severe courses of disease [19]. Furthermore, three exposures of vaccinations have been shown to effectively neutralize Omicron [16] which eventually results in less severe courses due to rapid viral clearance. However, the majority of our patients had no detectable SARS-CoV-2-S antibodies after several vaccinations, suggesting that active immunization may not be the only reason for the more favorable outcome. T-cell immunity was not assessed in our study, but it has been shown that the response is similarly attenuated [21, 22]. The poor vaccination response despite a complete primary series of vaccines and booster vaccinations is in line with previous reports on solid organ transplant recipients [21, 22].

Passive immunization by mAbs was the first antiviral treatment available. Dependent on the VOC mAbs were effective to a varying degree. Recently, we demonstrated in a cohort of lung transplant recipients with COVID-19 between the beginning of the pandemic and end of 2021 that the early application of casirivimab/imdevimab was associated with a significant survival benefit [7]. With Omicron casirivimab/imdevimab became ineffective. Another mAB sotrovimab was reported of having neutralizing activity in-vitro against Omicron and became available. Sotrovimab was the most frequently applied antiviral therapy in the current analysis. After increasing evidence on the efficacy of remdesivir in early COVID-19 [23] it became standard of care in many transplant centers. Additionally, in the beginning of our study molnupiravir became available and a beneficial side effect profile in transplant recipients was anticipated [15]. In contrast nirmatrelvir/ritonavir a highly CPY3A4-dependent drug leads to significant drug interactions [24] which likely was the main reason for sparse use in our study.

In a recently published publication UK-wide retrospective analysis in 142 SARS-CoV-2 (96% attributed to Omicron) infected kidney transplant recipients, 33% were treated with early Sotrovimab and 15% with early Molnupiravir. Admission rate was 21% in patients without early treatment and 1.4% died without early treatment. Use of sotrovimab was associated with lower admission rate (2%) indicating prevention of progression to severe disease in this study. In contrast to our study, early antiviral treatment was used less frequently (48 vs 76%) and severe and critical disease was reported infrequently in kidney transplant patients [25].

We focused on early treatments since antiviral therapy is most effective when viral replication takes place. Whether current or future antiviral measures in immunocompromised patients with prolonged and recurrent viral replication are of use was beyond the scope of our study. Furthermore, only early antiviral therapy was analyzed since we aimed to exclude a selection bias of patients in whom treatment was initiated when a severe course of disease was already present.

In the current analysis, early antiviral therapies seemed not to impact the course of COVID-19 significantly. This must be regarded with all limitations of our study. However, in contrast to the beneficial effect of casirivimab/imdevimab in lung transplant recipients with COVID-19 before Omicron becoming the dominant variant as reported previously [7], currently we were not able to demonstrate a similar association. Whether the neutralizing efficacy of sotrovimab for Omicron, or other Omicron specific factors are responsible for these observations remains unclear. Similarly, no association of early application of remdesivir and molnupiravir or combinations of different antiviral drugs with reduced odds for severe or critical courses of COVID-19 was found. In the setting of high degree immunosuppression with prolonged viral replication short-term antiviral attempts might, therefore, not be sufficient.

While sotrovimab retains in vitro activity against BA.1, a reduced activity against BA.2 has been debated [26–28]. It is currently unknown how this affects in vivo effectiveness of sotrovimab against BA.2. In multiple regression analysis, subvariant BA.1 or BA.2 was not associated with outcome, while rates of sotrovimab were similarly high.

Age and chronic kidney failure were independently associated with a worse outcome. Lung transplant recipients with a GFR < 30 ml/min/1.73m^2^ had three times increased risk for a severe or critical disease, highlighting the impact of kidney function on immune system in already immunocompromised patients. Age and comorbidities are known to be associated with worse outcome in patients with COVID-19 [29].

The results should be interpreted within their obvious limitations. This was a retrospective study with various antiviral therapies and combinations thereof applied. This is significantly in contrast to RCTs which are the standard to assess the efficacy of a therapeutic strategy. Our study should not change the present strategies to promote vaccination and should not prevent patients from getting antiviral therapies. Furthermore, the rapidly evolving field of COVID-19 makes a timely analysis difficult. The moving landscape of Omicron sublineages with the appearance of BA.4 and BA.5 and the increasing use of pre-exposure prophylaxis with tixagevimab and cilgavimab could not be addressed in this study. However, we believe that our observations on the impact of antiviral measures have highlighted the need for further preventive and therapeutic strategies. Course of COVID-19 was the primary outcome of the current study. In survivors, long-term sequela would be of interest but were beyond the scope of the study. Long-COVID and the impact of COVID-19 on CLAD development remain to be assessed.

In conclusion, our study demonstrates that in lung transplant recipients COVID-19 due to Omicron (B.1.1.529) has resulted in less severe cases and reduced mortality compared to previous variants. However, COVID-19 remains a significant threat for lung transplant recipients. Vaccination status with poor response and currently available early antiviral treatments were not associated with a reduced risk for severe or critical COVID-19. Advanced age and chronic kidney failure are risk factors for worse outcome. Additional antiviral measures are required for affected individuals.

## Data Availability

Data are available on reasonable request from the corresponding author.

## Abbreviations

BAU: Binding antibody units
CLAD: Chronic lung allograft dysfunction
COVID-19: Coronavirus disease 2019
GFR: Glomerular filtration rate
mAb: Monoclonal antibody
OR: Odds ratio
PCR: Polymerase chain reaction
SARS-CoV-2: Severe acute respiratory syndrome coronavirus type 2
VOC: Variant of concern
